# Seminal profile of 23,504 patients over the years: 7 years of experience

**DOI:** 10.5935/1518-0557.20180055

**Published:** 2018

**Authors:** Camila Nogueira, Ivan Henrique Yoshida, Fabia Lima Vilarino, Waldemar Pereira de Carvalho, Emerson Barch Cordts, Caio Parente Barbosa

**Affiliations:** Instituto Ideia Fértil de Saúde Reprodutiva, Santo André - SP, Brazil; 2 Faculdade de Medicina do ABC, Santo André - SP, Brazil

**Keywords:** seminal parameters, male infertility.

## Abstract

**Objective:**

To evaluate the behavior of seminal parameters over the years - 2010 to May 2017.

**Methods:**

A retrospective study, carried out from January 2010 to May 2017, covering men who underwent sperm examination. Seminal parameters (volume, sperm concentration, motility, morphology, age and duration of infertility in years) of 23,504 men were evaluated. The groups were compared to each other to check whether there were changes to the seminal parameters in that period.

**Results:**

There was no change over the years considering the time of infertility, in an average of 3.78 years. The mean age of the patients was 36.53 years, with a trend of increase in 0.2 years. In relation to the volume of semen samples, the mean value was 3.29mL, with a decrease trend in 0.05mL. As for sperm concentration, the average was 34.37 million/mL, with a decrease trend of 1.0 million/mL. Progressive sperm motility showed an average of 47.27% and there was a decrease trend of 0.67%. Finally, sperm morphology presented an average of 2.79% of normal spermatozoa, with a decrease trend of 0.33%.

**Conclusion:**

It can be noted that over the years, the sperm quality of men looking for assisted reproduction clinics has tended to decrease in macroscopic and microscopic parameters (volume, sperm concentration, motility and morphology).

## INTRODUCTION

The need for assisted reproduction technology (ART) procedures for the establishment of pregnancies has steadily increased worldwide (Anbari *et al*., 2016). Fertility peaks and then decreases over time in both men and women, thus the reproductive timeline may be one aspect to consider when determining the ideal time to start a family (Sharma *et al*., 2013).

Male infertility is a common and complex problem affecting 1 in 20 men. Global rates of male infertility range from 2.5% to 12%. This means that at least 30 million men worldwide are infertile (Agarwal *et al.*, 2016). Despite voluminous research in this field, in many cases, the underlying causes are unknown (Dada *et al*., 2012).

Semen analysis is the main evaluation of a man's fertility ability, and the analysis represents a picture of spermatogenesis (Neves *et al*., 2011). Semen parameters, such as sperm concentration, motility, viability, and morphology provide useful insight into the semen quality. However, the role and the correct interpretation of these semen parameters remain unclear and their implications on recurrent pregnancy loss are debatable (Ruixue *et al*., 2013).

As men age, testosterone levels begin to decrease, resulting in hypogonadism. However, if testosterone is used to treat hypogonadism, it can suppress spermatogenesis (Stewart & Kim, 2011). Semen parameters also begin a steady decline as early as 35 years of age (Dunson *et al*., 2004); semen volume and motility decrease, and morphology may become increasingly abnormal (Kimberly *et al*., 2012). After the age of 40, men can have significantly more DNA damage in their sperm, as well as decline in both motility (40%) and viability (below 50%) (n=504, *p*<0.001) (Varshini *et al*., 2012).

Semen quality has been considered as one of the most sensitive indicators of the adverse effects of environmental pollution. In addition to the physical environment, semen quality may also be affected by other factors, such as age, occupation, cigarette smoking, and lifestyle (Tang *et al*., 2015). There are many factors which adversely impact semen parameters such as environmental issues, tight under garments - which raises the local temperature, life-styles, occupational hazards and sleep deprivation (Al-Turki, 2015).

The present study aimed at evaluating the semen parameters of a population covering all the patients that performed the spermogram at Instituto Ideia Fértil over the course of 7.5 years.

## MATERIALS AND METHODS

### Patients

Retrospective case-control study carried out from January 2010 to May 2017, covering all patients who performed semen analysis in the Instituto Ideia Fértil de Saúde Reprodutiva, through which we evaluated the following seminal parameters: volume, concentration, motility and morphology. In addition, the age of the patients and the duration of infertility (in years) were also evaluated.

The groups were compared. A total of 23,504 patients were evaluated, being 2,344 in 2010; 2,633 in 2011; 3,045 in 2012; 3,218 in 2013; 3,397 in 2014; 4,022 in 2015; 3,388 in 2016 and 1,457 patients until May 2017.

To check for variations in the seminal parameters over the years, all the seminal analyzes were performed following the parameters recommended by the World Health Organization, 2010.

### Statistical Analysis

Quantitative variables were described by median and respective confidence intervals, according to the normality of the data (evaluated by the Shapiro-Wilk test). Interquartile regression was used to analyze the variation of the indicators in relation to the year. The confidence level was 5%. The program used was Stata^®^ 11.0.

## RESULTS

Statistical analysis revealed that in relation to the age of the patients, there was an increase of 0.2 years during these 7.5 years. The mean age of the patients was 36.53 years. There was no variation in relation to the time of infertility. The average was 3.78 years.

In relation to the volume of the analyzed samples, there was a trend of 0.05mL decrease over the years. The mean volume of patients' seminal samples was 3.29mL.

Considering the microscopic parameters, sperm concentration showed a tendency to drop by 1 million/mL over the 7.5 years (mean of 34.37million/mL). Sperm motility decreased by 0.67% compared to progressive spermatozoa (mean was 47.27%). There was also a decline in sperm morphology of 0.33% of normal spermatozoa (mean was 2.79%) (Table 1).

**Table 1:** Results from the comparison of seminal parameters (2010 to May 2017).

Evaluated parameters	Median	Variation
Age	36.53	0.2
Infertility duration (years)	3.78	There was no variation
Volume (mL)	3.29	-0.05
Sperm concentration (millions/mL)	34.37	-1.00
Progressive spermatozoa (%)	47.27	-0.67
Sperm morphology (% of normal)	2.79	-0.33

## DISCUSSION

Time and again, various studies have been published supporting a decline in sperm quality or dismissing it. Analyses of retrospective data indicate that sperm counts may have declined in some parts of the world, but there seems to be geographical variations in semen quality (Kumar & Singh, 2015). A retrospective study of 9,168 cases (men ages 20 to 77) obtained from the Andrology and Reproduction Laboratory in Cordoba, Argentina for 10 years (1995-2004) showed a significant decrease in seminal volume, sperm count, motility, viability and normal morphology (Molina *et al*., 2010). Another study between 1996 and 2007 in the Sfax area of Southern Tunisia in a sample of 2,940 men in infertile relationships assessed the decline in semen quality over a period of 12 years (Feki *et al*., 2009). On the other hand, another similar study conducted between 2000 and 2010 among young Swedish men from the general population concluded that there is no evidence of time-related deterioration in semen parameters (Axelsson *et al*., 2011).

Some factors such as radiation, smoking, varicocele, infection, urinary tract infection, environmental factors, nutritional deficiencies and oxidative stress contribute to male infertility (Ahmadi *et al*., 2016). Spermatogenesis and maturation processes can be affected by fluctuations in hormones, temperature, dietary balance, and exposure to toxins due to habits or environmental pol lutants (i.e., smoking, alcohol, cadmium, lead, radiation, pesticide, endocrine disruptive chemicals) (Khatun *et al*., 2018).

**Figure 1:**
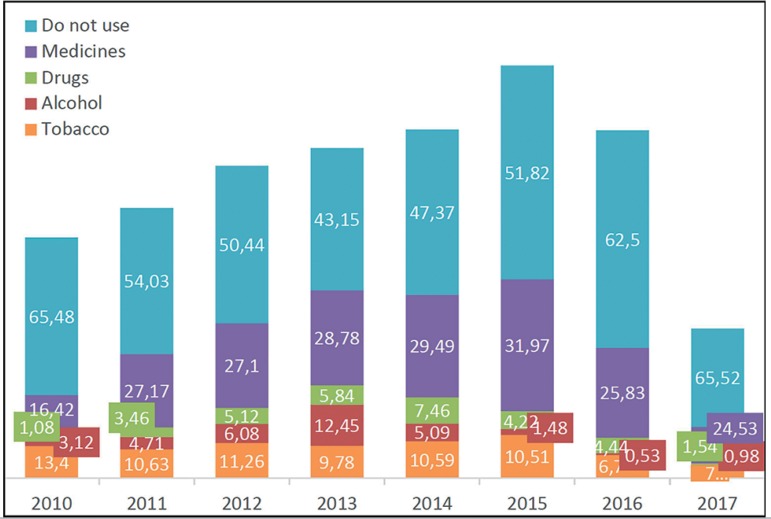
Comparative use of medications, drugs, alcohol and tobacco over the years.

Decreased general health status has been associated with lower sperm concentration, lower total testosterone levels and higher follicle-stimulating hormone values (Ventimiglia *et al*., 2015). Studies have also shown that many drugs are harmful to spermatogenesis and can lead to a temporary or permanent difficulty in conception (Guo *et al*., 2017; Brezina *et al*., 2012). These studies corroborate the findings among our patients at Instituto Ideia Fertil. It may be noted that in recent years there has been a decrease in the use of drugs, alcohol and tobacco, but the use of continuous medications has tended to increase (medications such as antidepressants, diuretics and insulin) (Figure 1).

In this study, we can note changes in seminal parameters over the years between 2010 and 2017. However, this result is based only on the population that sought our institution. Therefore, new analyses are necessary to generalize this result.

## CONCLUSIONS

Our data showed that there was no difference between the infertility duration in the men who sought the human reproduction service during the period from 2010 to 2017. However, there was an increase in the age of these patients, with reduction of seminal quality in all parameters evaluated (volume, concentration, motility, morphology).

These results are based only on the population that came to our institution. Further analyses are required to compare the results.
